# Grape and Wine Composition in *Vitis vinifera* L. cv. Cannonau Explored by GC-MS and Sensory Analysis

**DOI:** 10.3390/foods10010101

**Published:** 2021-01-06

**Authors:** Giacomo L. Petretto, Luca Mercenaro, Pietro Paolo Urgeghe, Costantino Fadda, Antonio Valentoni, Alessandra Del Caro

**Affiliations:** Dipartimento di Agraria, Università degli Studi di Sassari, Viale Italia 39/A, 07100 Sassari, Italy; gpetretto@uniss.it (G.L.P.); mercenar@uniss.it (L.M.); paolou@uniss.it (P.P.U.); cfadda@uniss.it (C.F.); avalentoni@uniss.it (A.V.)

**Keywords:** *Vitis vinifera* L., berry quality, grape, wine, VOCs, sensory analysis

## Abstract

GC-FID/MS is a powerful technique used to analyze food and beverage aromas. Volatile organic compounds (VOCs) in grape berries play an important role in determining wine quality and are affected by many factors, such as climate and soil that mainly influence their relative concentrations. Wine aroma is generated by a complex mixture of compounds, and the sensory relevance of individual VOCs is far from elucidated. Herein, the VOC content (free and glycosylated) of Cannonau grape skin and juice and of Cannonau wine collected in different areas of Sardinia is explored. Wine sensory analysis was also carried out and the relationship between sensory attributes and VOCs was investigated. Although Cannonau grapes showed the same VOC fingerprint, great variability was identified between samples, although only the differences in 2-phenylethanol and benzyl alcohol concentration in the grape skins and benzyl alcohol and a terpenoid in grape juice were significantly different according to ANOVA. The correlation between VOC content and the sensory profile highlights the role played by 2-methyl-1-butanol and 2-phenylethanol in increasing wine sensory complexity.

## 1. Introduction

High-quality wines derive from high-quality grapes. Wine quality is affected by a variety of factors, such as the vineyard’s location, soil, climate, and topography [1]. To date, the concept of “terroir”, which for many years has represented the relationship between a wine’s sensory profile and its geographic origin [2], includes many natural and human factors, such as geographical location, soil, topography, climate, exposure to solar radiation, and the viticultural and enological practices applied [3,4].

Wine is one of the most popular beverages all over the world, and two key parameters are closely monitored by winemakers to ensure that customer expectations are satisfied: production costs and consumer sensory satisfaction. The chemical composition of wine is greatly variable. It is dominated by water (which attributes approx. 85%) and then ethanol (about 12%) produced by yeasts during the fermentation process. In addition to these two main components, many minor compounds are present making up the remaining ~3% of the chemical composition. These minor components, primarily phenolic and volatile compounds, are considered to contribute the most to the quality of the wine. All these substances are influenced by a multitude of factors, such as the genetic specificity of the grape variety, the environmental conditions, grape growing techniques, and—of utmost importance—the winemaking processes [1,5]. 

Volatile organic compounds (VOCs) are considered to be the most important attributes contributing to the sensory properties of wine. They are classified according to their source or origin: primary compounds, also known as varietal compounds, are derived from the grapes themselves; secondary compounds arise from yeast metabolism; and post-fermentative compounds arise from chemical reactions that take place during the wine’s ageing process [6]. Primary compounds may occur in free as well as in bound forms, depending on the odor active molecule (aglicone) that may, or may not, be bound to a sugar moiety. Bound forms are commonly known as aroma precursors since they undergo hydrolysis easily, generating the active odor molecule and free sugar. Some grape varieties are characterized by high concentrations of odor-active forms and are classified as “floral” varieties; for example, Muscat varieties are characterized by large amounts of free terpenoids. By contrast, when terpenes in the free form occur at low concentrations (in general lower than the respective odor threshold), these varieties are classified as “non-floral” varieties, such as Chardonnay.

Several classes of VOCs have been detected in grapes. Of these, the main varietal compounds are: terpenes (mainly present in grape skin) [7], methoxypyrazines [8] (carotenoid-derived compounds also known as norisoprenoids) [9], thiols [10], benzene derivatives [11], and compounds derived from lipid oxidation [12]. The production of secondary metabolites in the grape—and therefore their contribution to the chemical composition of the wine’s aroma—is greatly variable and strongly influenced by the “terroir” [13] as well as by agronomic practices [14]. In recent years, many authors have studied the effects of agronomic practices and of the interactions of the vine with the soil and climate on the VOCs, with the aim of being able to modulate and improve the chemical composition of the grape’s aroma [14,15]. Several attempts have been made, in the last ten years, to relate the wine aroma profile to sensory data but often the results obtained are weak or difficult to explain due to different reasons, namely the statistical method used to explore this relationship or the use of an imprecise or not clear sensory terminology [16]. Moreover, it is difficult to fully understand the contribution of the varietal composition alone to the overall wine flavor. In fact, many interactions occur in wine between the varietal aromas and other nonodorant wine components, that influence the wine sensory characteristics [6].

“Cannonau di Sardegna” is a red wine, designated a DOC (Controlled Designation of Origin) wine since 1972 [17], and produced by the homonym variety typical of Sardinia. Based on the ampelographic data and genetic observations, the Cannonau grape has been linked to Grenache noir cultivated in France, and Garnacha tinta cultivated in Spain [18,19]. According to this classification, Grenache is cultivated all over the world, and it has been estimated that the total area cultivated with this grape variety is little short of 200,000 hectares [20]. In Europe, France and Spain are the predominant producers, cultivating approx. 81,000 and 62,000 hectares, respectively. Italy is ranked third in Europe for the cultivation of this grape, with a dedicated ~8000 hectares, 7600 of which are in Sardinia. Although the worldwide cultivated area of Grenache has been decreasing since 2004 [21], this variety, which adapts well to hot and dry regions, is well known in France, Spain, and Sardinia for the very good, interesting wines it produces.

Previous studies conducted on Grenache in Spain suggest that the soil may affect wine chemical composition and quality. De Andrés-de Prado et al. [22] reported that wines obtained from grapes grown in richer soil and with a minor coarse fraction presented a lower total phenolic content and color intensity, but a higher stilbene concentration [22]. Moreover, the cultivation site can influence the quality of Grenache grapes. In an experimental trial carried out in Terra Alta (Catalonia, Spain) investigating optimal grape ripeness in two different terroirs—named “early” and “late” ripeness terroirs (in reference to their usual date of harvest), characterized by warm and temperate climates, respectively—Grenache grapes from late terroirs were able to maintain higher levels of acidity—assuring wine quality—compared with those cultivated in early terroirs [23].

In relation to Cannonau in Sardinia, the first study assessing the volatile fraction of Cannonau wine entailed grapes cultivated in a single restricted area: the Jerzu area (NU) of central Sardinia [24]; the study was specifically focused on assessing the effects of different agronomic practices and different winemaking technologies on VOCs, which were only assessed qualitatively [24]. More recently, commercial Cannonau wines have been evaluated for their antioxidant activities and vasodilatory properties [25], as well as their content of free amino acids and biogenic amines [26]. Cannonau grape morphological and agronomical parameters have also been characterized on 85 Cannonau biotypes cultivated in a single vineyard in south-west Sardinia [27].

Therefore, our knowledge about the VOC content of berry skins and juice from “Cannonau di Sardegna” is very limited. Finally, the sensory aspects of Cannonau have never been studied in any depth and no studies have reported on the possible correlations between volatile composition and sensory evaluations of the wines obtained from the Cannonau variety.

Hence, the aim of the present study was to investigate the volatile chemical composition of Cannonau grapes collected in different areas of Sardinia (the primary Italian region for Cannonau cultivation) and their relative wines. Both qualitative and quantitative analyses of free and bound volatiles from grapes were investigated by means of GC-FID/MS. Moreover, in order to provide a first insight into the relationship among the Cannonau volatile composition and sensory descriptive analysis the presence of any correlations between volatile composition and sensory attributes used to describe this red wine was explored, with the aim to contribute to fill the gap of knowledge on this important worldwide variety.

## 2. Materials and Methods

### 2.1. Reagents and Chemicals

Ultrapure 18.3 MΩ⋅cm water used for these experiments was produced from a Zeneer Power III TOC system (Human Corp., Seoul, Korea). All the following reagents were purchased from Sigma Aldrich, unless stated otherwise, and were standard grade: butyl acetate, hexanal (Alfa Aesar, Haverhill, MA, USA), butanol, 3-methyl-1-butanol (Alfa Aesar), (E)-hex-2-enal (Alfa Aesar), hexanol, (Z)-hex-3-en-1-ol (Alfa Aesar), nonanal, 2-ethyl-1-hexanol, acetophenone, α-terpineol, benzyl alcohol, 2-phenylethanol, ethyl tetradecanoate, ethyl dodecanoate, methyl 2-hydroxybenzoate (Alfa Aesar), citronellol, ethyl decanoate, benzaldehyde (Alfa Aesar), ethyl octanoate, ethyl heptanoate, isoamyl acetate, limonene, 2-methyl-1-butanol, ethyl hexanoate, ethyl acetate, ethyl butanoate, Saccharomyces cerevisiae (Franke, Italy), (NH_4_)_2_HPO_4_ (Franke, Italy), K_2_S_2_O_5_ (Franke, Italy), tartaric acid, polyvinylpolypyrrolidone (Alfa Aesar), sodium azide (Alfa Aesar), NaOH, Na_2_PO_4_(H_2_O)_2_, citric acid, HCl, 3-octanol, MeOH, CH_2_Cl_2_, β-glucosidase enzyme (Ecozim AROM, CRC Biotek, Roma, Italy), hydrocarbon mixture from C8–C23 (Sigma-Aldrich, St. Louis, MO, USA).

### 2.2. Grape Materials

Fourteen samples of Cannonau grapes at technological maturity were collected in 2018 from different vineyards located across Sardinia: 6 samples came from northern Sardinia (NS; Alghero, Sorso, and Usini in the province of Sassari); 7 samples came from central Sardinia (CS; Dorgali, Oliena, and Orgosolo in the province of Nuoro); and 1 sample from southern Sardinia (SS; Serdiana in the province of Cagliari). The different number of samples from the different Sardinian terroirs depended on the interest expressed by the wineries to be involved in this project, reflecting the different levels of importance that Cannonau wine has in the three areas of cultivation. Cannonau grape is the most diffuse cultivar grown in the vineyards of central Sardinia, whereas in northern Sardinia, the white grape cultivar Vermentino is very popular, being cultivated at comparable levels. Finally, in southern Sardinia, other red grape cultivars, such as Bovale, Monica, and Carignano in particular, are more popular and more extensively grown than Cannonau. The grape musts were analyzed for sugar content, pH, and total acidity (gL^−1^ of tartaric acid) according to the Official European Methods of Commission Regulation EEC N° 606/2009.

The year 2018 was characterized by a high rainfall with respect to the average, with the months from June through to September recording the highest levels of precipitation for the last 96 years. With regard to temperature, July was the hottest month of the year with a maximum temperature of 35 °C [28].

The investigated vineyards in the northern part of Sardinia are located at altitudes ranging between 50 and 250 m a.s.l. The soils are characterized by a sandy-clay texture or clay-limestone texture and soil depth varies from 0.8 to 1.2 m. In general, the soils are fertile and rich in nutrients and organic matter, with moderate permeability and drainage, but with a large water reserve. The average annual rainfall for the area (considering the last 30 years) is about 545 mm, but 725 mm were recorded in 2018. In this growing area, the vines are Vertical Shoot Position (VSP) trained, spur or cane pruned, and have a plantation density that ranges from 3200 to 4500 vines ha^−1^. The vines are trained on wire to 80 and 110 cm above the soil, and the canopy normally exceeds one meter. The vineyards are irrigated, and the soil is cover cropped but no vineyard was irrigated in 2018.

Regarding the vineyards located in the central part of Sardinia, their altitudes range from 250 (Dorgali) to 700 m a.s.l. (Orgosolo). Sun exposure is always to the southeast, and the soil origin is mainly derived from granitic materials. The soils are poor with a low content of organic matter and nutrients, and they have a very low aptitude for retaining water. In this area, rainfall (30 years average) ranges from 620 mm close to the sea (Dorgali) to 740 and 790 mm over the mountains (Oliena and Orgosolo, respectively). The annual rainfall for the 2018 vintage was 1220 mm in Dorgali, 1180 mm in Oliena, and 1290 mm in Orgosolo. The planting densities of the vineyards vary between 4000 and 6500 vines ha^−1^. In this growing area, vines are traditionally trained according to the little bush system. However, in the seven participating vineyards of this study, vines are trained using a VSP trellis system. Vines were not irrigated in 2018.

The only vineyard sited in southern Sardinia is located 100 m a.s.l. and characterized by a sandy-clay texture. The soil is fertile and deep with a good supply of organic substance and nutrients. This area of southern Sardinia is characterized by less rainfall compared with the regions hosting the other terroirs present in the island. The average rainfall for the last 30 years is 445 mm, whereas that for 2018 is 624 mm. The planting density is equal to 6500 vines ha^−1^. The vineyard is managed by a VSP system, spur pruned, on wire to 80 cm above the soil and the canopy normally exceeds one meter. The vineyard is irrigated but, also in this case, no water was provided in 2018.

Five samples from northern Sardinia, two from central Sardinia, and one from the south were also subjected to winemaking following the procedure reported below.

### 2.3. Winemaking Process

Grape samples NS1, NS2, NS3, NS4, NS5, CS1, CS2, and SS1, collected from the different areas (the specifications for each vineyard are shown in Table 1) were subjected to vinification using a standardized process to minimize effects due to technology. All these grape samples came from the most representative vineyards of the three different areas, NS, CS and SS, object of this study. Briefly, stems were removed from 50 kg of grapes by means of an automatic stainless-steel crusher destemmer (Enoitalia, Italia) and the must obtained was transferred into 100 L bins. The following were added to the musts: 10 g dry yeast (*Saccharomyces cerevisiae*, Franke, Italy), 15 g (NH_4_)_2_HPO_4_ (Franke, Italy), and 3.5 g hL^−1^ K_2_S_2_O_5_ (Franke, Italy). Then, musts were subjected to a 10-day maceration at controlled temperature (<28 °C), punching the cap down twice a day and measuring must temperature and sugars at the same time to monitor alcoholic fermentation. Fermentation stopped when the sugar content reached 0, measured in Babo units. To separate the must from the marcs, a 40 L hydraulic press (Grifo Marchetti, Italy) was used at a maximum pressure of 2 bar and the resulting wine, corrected with sulfur dioxide, to achieve a value of at least 30 mg L^−1^ of free SO_2_, was transferred in a 1 hL stainless steel container and put in a cell at a temperature of 16 °C until the analyses. Wines were racked ten days after pressing, then K_2_S_2_O_5_ was again added to preserve wines from oxidation. Finally, after a second racking, performed two months later by the addition of K_2_S_2_O_5_, wines were subjected to sensory analysis.

### 2.4. Extraction of Free and Bound Volatile Compounds from Juice and Skin

The extraction of free and bound volatile compounds from the fourteen grape samples was carried out according to the method published by Genovese et al. [12]. Briefly, an extracting solution was prepared by adding 2.5 g tartaric acid, 5 g polyvinylpolypyrrolidone (PVPP) and 1 g sodium azide to 500 mL water. The pH was adjusted to 3.2 using NaOH 1N. A citrate/phosphate buffer was prepared by mixing 9.15 g Na_2_PO_4_(H_2_O)_2_ and 4.67 g citric acid in 500 mL water. The final pH was adjusted to 5.5 using HCl or NaOH.

The skins from 250 g of grapes, exactly weighed, were extracted for 24 h at room temperature using 250 mL extracting solution. The resulting solution was then centrifuged at 9000 rpm for 20 min at 18 °C. An amount of 50 mL of the supernatant was spiked with 100 µL of an internal standard solution (3-octanol in MeOH 225 mg L^−1^) and passed through C18 extraction column (1 g, UCT Clean-Up^®^), which was preconditioned according to the manufacturer’s instruction. The column was then washed with 10 mL water and the loaded free volatile compounds were eluted with 5 mL CH_2_Cl_2_; they were then dried with anhydrous sodium sulfate, concentrated to about 50 µL under nitrogen flow, and analyzed by GC-FID/MS. The glyco-conjugated compounds were eluted using 10 mL methanol and the resulting solution was then evaporated under vacuum at 35 °C until all the water was removed. The residue was then dissolved in 5 mL of the previously described citrate/phosphate buffer. Then, 60 mg of β-glucosidase enzyme (Ecozim AROM, CRC Biotek) were added to the residue, which was incubated at 40 °C for 16 h [12]. The resulting mixture was spiked with 100 µL of internal standard solution (3-octanol in MeOH, 225 mg L^−1^) and passed through a C18 extraction column (1 g, UCT Clean-Up^®^) preconditioned according to the manufacturer’s instructions. The column was then washed with 10 mL of water and the loaded volatile compounds resulting from the enzymatic hydrolysis were eluted with 5 mL CH_2_Cl_2_. The resulting solution was dried with anhydrous sodium sulfate, concentrated to about 50 µL under nitrogen flow, and analyzed by GC-FID/MS. The juice obtained by hand pressing 250 g of grapes was subjected to the same treatments adopted for the skin extracts, as above reported.

### 2.5. Grape VOC Gas Chromatography (GC-FID/MS) Analysis

The volatile organic compounds extracted were subsequently analyzed in a 7890 GC equipped with a Gerstel MPS autosampler. The chromatographic separation was performed on a VF-Wax column 60 m × 0.25 mm i.d., 0.5 µm film thickness column (Agilent). The following temperature program was used: 40 °C hold for 4 min, then increased to 150 °C at a rate of 5.0 °C/min, held for 3 min then increased to 240 °C at a rate of 10 °C/min, and finally held for 12 min. Helium was used as the carrier gas at a constant flow of 1.8 mL/min. At the end of the column, the flow was split into an Agilent 7000C MSD detector and a Flame Ionization Detector (FID) at a ratio of 2:3; this was achieved by means of a Gerstel µFlowManager µsplit 2-way. The data were analyzed using a Mass Hunter Workstation B.06.00 SP1, and identification of the individual components (Table 2) was performed by comparison against co-injected pure compounds and by matching the MS fragmentation patterns and retention indexes using the built-in libraries, literature data, or commercial mass spectral libraries (NIST/EPA/NIH 2008; HP1607 purchased from Agilent Technologies). 

The quantification of compounds was carried out using the internal standard method (3-octanol) on FID chromatogram. Each standard was accurately weighed and dissolved in 10 mL ethanol; the resulting stock solution was diluted with ethanol and the internal standard was added in order to obtain five levels according to the linearity range reported in Table 2. Compounds who gave well-resolved peaks were quantified. For some compounds, whose standards were not available in the laboratory, quantification was performed using the calibration curve for a compound of the same classes of volatiles as reported in Table 2 [29]. Results were expressed as µg kg^−1^ of grape.

A hydrocarbon mixture of n-alkanes (C8–C23) was analyzed separately under the same chromatographic conditions used on the VF-Wax capillary columns to calculate the retention indexes using the generalized equation by van Den Dool and Kratz [30].

### 2.6. SPME Conditions for Wine VOC Analysis

The wines were subjected to solid phase micro extraction (SPME) coupled with gas chromatography in order to obtain the VOCs chemical composition according to a previously optimized method (see Supplementary Materials).

The isolation of headspace volatile compounds was carried out using a 100 μm PDMS/DVB/CAR (Polydimethylsiloxane/Divinylbenzene/Carboxen) coated fiber (Supelco, Sigma Aldrich, St. Louis, MO, USA) that was preconditioned according to the manufacturer’s instruction. An amount of 10 mL wine and 100 µL internal standard (3-octanol, 225 mg L^−1^) were placed into a 20 mL SPME vial (75.5 × 22.5 mm) that was tightly closed using a septum. After 5 min of equilibration at 60 °C, the conditioned fiber was injected through the septum and suspended in the headspace.

The fiber was exposed to the volatiles for 30 min; it was then retracted, removed from the vial, and placed immediately into the injector of the GC. Thermal desorption was performed in the injector at a temperature of 250 °C for 5 min in splitless injection mode. Prior to and after each analysis, the fiber underwent a further bake-out step for 5 min at 250 °C.

### 2.7. Wine VOC Gas Chromatography-Mass Spectrometry (GC-MS) Analysis

The volatile organic compounds absorbed by the fiber were subsequently analyzed in a 7890 GC equipped with a Gerstel MPS autosampler and coupled with an Agilent 7000C MSD detector. The chromatographic separation was performed as previously described in the section entitled “Grape GC-FID/MS analysis”. A hydrocarbon mixture from C8–C23 was injected under the same HS-SPME/GC-MS conditions to obtain the linear retention indexes.

The quantification of compounds in the headspace was carried out using the internal standard method (3-octanol). Each standard was accurately weighed and dissolved in 10 mL ethanol; the resulting stock solution was added to internal standard and diluted with ethanol:water (12:78 *v*/*v*) to obtain five concentrations according to the linearity range reported in Table 2. The pH of each sample was adjusted to 3.5 by the addition of tartaric acid. Results were expressed as µg L^−1^ of wine.

### 2.8. Sensory Analysis

A quantitative descriptive analysis (QDA) was performed using a trained panel of 14 oenologists (aged between 23 and 27 years). The sensory ballot was adapted from a ballot used to describe wines obtained from Grenache grapes and already verified for the Cannonau sensory profile [31]. The eight samples of Cannonau from the different areas were labeled with three-digit code numbers and presented, at room temperature, to the panel in monadic sequence and in a randomized and balanced order to avoid carry-over effects. Each attribute was quantified using a structured scale from 0 to 9, where 0 was not perceptible and 9 was strongly perceptible. The attributes analyzed were the following: color intensity, olfactory intensity, odor (rose, cherry, jam, dried plum, herbaceous), taste intensity, acidity, bitterness, astringency, body, flavor (rose, cherry, jam, dried plum, herbaceous), persistency, and aftertaste.

### 2.9. Statistical Analyses

Statistical analysis was carried out using the statistical package Statistica 10 for Windows (StatSoft). To test the normality of the data set of the VOCs, skewness and kurtosis were performed. Most of the data were normally distributed. For this reason, one-way ANOVA was performed on the grape juice and skin volatile composition and on wine sensory data to find differences between the samples. Mean data, where required, were separated using the LSD test (*p* < 0.05). The entire data set was also analyzed by using a non-parametric method, the Kruskal–Wallis one-way analysis of variance, which does not assume a normal distribution of the residuals. The results obtained from the application of the two different methods (parametric and non-parametric) were identical. Principal component analysis (PCA) was performed on the volatile compounds and sensory data to extract the most relevant information [32], followed by a Pearson correlation (*p* < 0.05) to highlight any significant relationships between the data. Finally, Partial Least Squares (PLS) regression was applied to determine the relationship between the predictors (independent variables: volatile compounds) and the responses (dependent variables: sensory attributes).

## 3. Results and Discussion

### 3.1. Grape Technological Parameters at Maturity 

The following values are the overall means obtained for the grape samples collected from the vineyards located in the three distinct Sardinian areas investigated (NS, CS, and SS, respectively): sugar content (expressed in °Brix): 23.9 ± 1.9, 24.3 ± 1.8, and 24.8 ± 0.1; total acidity (expressed in g L^−1^ of tartaric acid): 3.6 ± 0.6, 3.9 ± 0.8, and 3.5 ± 0.06; and pH: 3.68 ± 0.2, 3.57 ± 0.1, and 3.53 ± 0.06.

### 3.2. Volatile Aroma in Grape Juice and Skin

The qualitative analysis of VOCs extracted from the Cannonau grapes showed the presence of 38 compounds (free and bound) in the grape juice and skins (Table 2). Some of the classical categories of compounds commonly detected by GC/MS in grapes were also found in the Cannonau grape. The compounds deriving from the oxidation of fatty acids were well represented by the aldehydes, hexanal and hexenal (in the free form only) and their respective alcohols, which were detected in both the free and bound forms. In addition to the compounds deriving from lipid oxidation, shikimic acid pathway derivatives were also found, including benzyl alcohol, 2-phenylethanol, and benzaldehyde. With regard to terpenes, in addition to limonene and α-terpineol, found in both the juice and skins, the GC/MS analysis showed an additional peak with a retention index of 1424 and a fragmentation pattern typical of terpenoids (136, 121, 110, 95, 93), but that could not, unfortunately, be identified. The only compounds detected in Cannonau grapes attributable to the class of “heavy sulfur compounds” were benzo[*d*]thiazole and 3-(methylthio)propan−1-ol, which were detected in the juice and skins.

Table 3 and Table 4 report the concentration ranges of free and bound compounds, respectively, and the average value for each compound detected and quantified in the samples collected in the three areas, NS, CS, and SS. The first insight obtained from the quantitative results was the presence of great variability within the samples. Quantitative data of VOCs in Cannonau grape juice and skins, obtained by internal standard calibration curves of FID chromatograms, showed there to be no significant differences among the three areas, NS, CS, and SS, with the exception of two free compounds present in the skin—benzyl alcohol and 2-phenylethanol—and two compounds—benzyl alcohol and a terpenoid— present in the juice, as shown by ANOVA (Table 3).

As observed, the most abundant volatiles, hexanal, (*E*)-hex-2-enal, hexanol, (*Z*)-hex-3-en-1-ol, and (*E*)-hex-2-en-1-ol, were the products of lipid oxidation (Table 3). The C6 compounds are considered responsible for the green, herbaceous, and vegetable aromas in wine [33]. Except for nonanal, also present in the bound form (Table 4), which could arise from the degradation of linolenic acid [11], other C6 compounds (aldehydes and alcohols) were only detected in free forms. One study in the literature has reported the presence of C6 alcohols in both the free and bound form [34], whereas others [12], in agreement with our results, report the absence of C6 aldehydes in the bound form.

The main monoterpene detected in Cannonau grapes was α-terpineol, ranging from 2.6 to 15.7 µg kg^−1^ and 0 to 20.0 µg kg^−1^ in skins; and 0.04 to 14.6 µg kg^−1^ and 0.9 to 14.6 µg kg^−1^ in juice for the free and bound forms, respectively. In line with a previous study [35], the glycosylated monoterpenes were found to be more abundant than the respective compounds in the free form. The average total amount of terpenoids (α-terpineol, limonene, and the unidentified terpenoid) present in grape skins accounted for 28.6 µg kg^−1^ (free and bound forms combined) in the NS samples, and 31.1 µg kg^−1^ (free and bound forms combined) in the CS samples. Regarding the grape juice, the total terpenoid content accounted for 25.07 µg kg^−1^ (free and bound forms combined) in the NS samples, and 35.9 µg kg^−1^ in the CS samples. With regard to the SS grape sample, the sum of the terpenoids in the skins accounted for 14.81 µg kg^−1^ (free and bound forms combined), and in the juice the total content was 43.64 µg kg^−1^. As known, the concentration of terpenes in grapes is affected by their sun exposure. Several authors have studied the effect of light on terpene concentration in grapes [36], and the general conclusion that emerges is that the concentration of monoterpenes increases according to the level of sun exposure [14]. Moreover, Ubeda et al. [37] have highlighted the role of grape maturity on the content of glycosylated terpenes, which tend to increase during maturation. In our case, the SS sample was characterized by the highest sugar content with respect to the NS and CS samples, in agreement with the literature data.

The benzene derivatives identified in the studied samples were represented by benzaldehyde, benzyl alcohol, and 2-phenylethanol. The latter is a derivative of phenyl propanoid metabolism and produces a rose-like aroma. This compound was absent in the bound volatiles of the juice, whereas it ranged from 12.5 to 34.3 µg kg^−1^ and from 0.0 to 10.3 µg kg^−1^ in the skin (free and bound forms, respectively), and from 7.7 to 23.5 µg kg^−1^ in the juice (free form only). The concentration of benzyl alcohol (free form) was significantly higher (*p* < 0.05) in the skin and juice samples from the north of Sardinia with respect to the samples collected in central Sardinia (Table 3). The highest value was reported for the SS grape juice (81.8 µg kg^−1^). The same trend (*p* < 0.05) was also observed for the free form of 2-phenylethanol in the skins. This latter result could suggest a slight influence of the terroir on the shikimic acid biosynthetic pathway of benzene derivatives. As reported by Kalua and Boss [11], their concentration increases during the final grape maturation step. Moreover, it was recently reported that foliar fertilization with an aqueous water solution of phenyl alanine increases the concentration of some benzene derivatives [38], confirming that the common intermediate in the reaction pathway of the biosynthesis of benzenoids volatiles is the amino-acid phenyl alanine.

Although the results for the free and bound compounds (Table 3 and Table 4, respectively) reveal great variability between the samples in relation to the concentrations of individual compounds, as observed by the maximum and minimum values (with the exception of very few compounds), no significant differences (*p* ≥ 0.05) were found in the mean concentrations for the main group of volatiles between the regions investigated (data pertaining to southern Sardinia were not considered in the statistical analysis since only one sample was studied). This conclusion agrees with the results reported by Mendez-Costabel et al. [39], who studied the effect of seasonal variability and region of cultivation on the green aroma compounds present in *Vitis vinifera* L. Merlot in California. The authors detected great variability in the production of green aroma compounds, such as C6 compounds and methoxypyrazines, according to seasonal variability, whereas no significant variation was observed according to the region of vineyard cultivation. On the contrary, Ubeda et al. [37] found that the amount of alcohols, acids, and ketones in the Pais cv grape was influenced by vineyard location. The same occurred in relation to terpene content. In general, they observed that the bound aroma fraction was mostly influenced by location, whereas the level of grape maturity achieved had a greater influence on the free aroma profile. In reference to our data, we can observe that the sum of the VOCs detected in the skins (free and bound) for the three areas, NS, CS, and SS, were very similar: 2081.0, 2129.7, and 1825.1 µg kg^−1^, respectively. On the contrary, a big difference was found in the total amount of VOCs detected in the juice. Grapes cultivated in the SS area had a VOC content equal to 2959.5 µg kg^−1^, significantly higher compared with that for NS and CS at 2006.3 and 1892.2 µg kg^−1^, respectively. The SS sample had the highest content of free forms (skin and juice) at 3780 µg kg^−1^, compared with NS and CS samples at 3069.7 and 2717.2 µg kg^−1^, respectively. On the contrary, the sum of the bound forms (skin and juice) was very similar among the three areas, 1017.6, 1304.7, and 1004.4 µg kg^−1^, for NS, CS, and SS, respectively, confirming the findings of Ubeda et al. [37] on the role of location on the grape content of bound VOCs.

### 3.3. Volatile Aroma in Wine

Chemical characterization of wine volatiles was carried out by HS-SPME coupled with GC-MS analysis. The optimization of the two variables, extraction time and extraction temperature, for the fractionation of headspace wine volatile compounds was performed. For this purpose, a multivariate approach (fractional factorial design) was carried out (see supporting information, Table S1 and Figure S1), and a set of 13 extraction experiments performed. The ranges considered for the experiments were 40–60 °C for the temperature and 30–40 min for the extraction time, in accordance with the literature data [40,41]. The ratio between the areas of target compound and the area of internal standard was considered. The sum of these ratios for each class of compound (esters, alcohols, acids, and terpenes), analyzed using a linear model, generated four response surface areas.

The analysis of these four models (Figure S1) revealed that the extraction time in the range considered had less effect with respect to the temperature for three of the four classes. This evidence led us to select an extraction time of 30 min and an extraction temperature of 60 °C.

The qualitative chemical characterization of wine volatiles (Table 5) showed that the headspace of Cannonau wines was homogenous among the samples analyzed. In fact, the general VOC fingerprint was very similar for all the samples studied. The GC-MS analysis revealed the presence of 37 main components and, in addition to several varietal compounds already detected in Cannonau grape samples, several volatiles that arise from yeast metabolism were identified. Limonene, hexanol, citronellol, and other compounds can all be classified as varietal compounds, whereas the two isomers of methyl-butanol and the ethyl esters of fatty acids are commonly linked to yeast metabolism. The volatile esters produced by yeasts are commonly classified as acetate esters and medium-chain fatty acid esters. Both these categories are well represented in Cannonau wines. A comparison of our data with the results of Begala et al. [24] revealed some similarities among the classical compounds detected in wines. Beside the typical ethyl esters of fatty acids, the varietal compounds limonene, 2-ethyl-1-hexanol, benzaldehyde and benzyl alcohol were also detected in Cannonau samples from Jerzu [24]. The quantitative data, obtained using calibration curves of pure standards (Table S2), showed a predominance of yeast-derived compounds in the headspace (Table 5). Of all the quantified compounds, the two isomers of methyl butanol (mean value 132,196 µg L^−1^, obtained by summing all the samples) and 2-phenylethanol (mean value 56,402 µg L^−1^, as above) were the main volatiles in the headspace. In the classical Erlich pathway, these components arise from amino acids leucine/isoleucine for methyl butanol isomers and from phenylalanine for 2-phenyl ethanol; their sensory attributes are banana/fruity and rose/flower, respectively.

Ethyl acetate was the main volatile ester present belonging to the group of acetate esters. Overall, the acetate esters are produced in much higher concentrations with respect to medium-chain fatty acid esters, and therefore much attention is usually given to these compounds. In the Cannonau wines, ethyl acetate ranged between 18,088 µg L^−1^, for the NS3 sample, and 33,049 µg L^−1^, for SS1. Its odor threshold in wine has been estimated to be 7500 µg L^−1^ [42], much lower than the concentration detected, thus suggesting it to make a consistent fruity/solvent-like contribution to the sensorial characteristic of the wine. Like ethyl acetate, isoamyl acetate is also included in the acetate ester group. This compound has been detected at concentrations (mean value 119 µg L^−1^) greater than its estimated odor threshold of 30 µg L^−1^ [42]. Isoamyl acetate contributes a banana/fruity attribute to the flavor. Among the medium-chain fatty acid esters, the main compounds were found to be the ethyl esters of butanoic, hexanoic, and octanoic acid. Their measured concentrations (mean values: 265, 710, and 136 µg L^−1^ for butanoic acid, hexanoic acid, and octanoic acid ethyl esters, respectively) were always higher than their respective odor thresholds [43]. The comparative analysis of samples showed that the samples from north Sardinia and south Sardinia differed with respect to those of central Sardinia in the concentration of ethyl-hexanoate and ethyl-octanoate both lower in the samples CS1 and CS2 (*p* < 0.05). The same trend was also observed in the concentration of ethyl butanoate although the statistical analysis did not find any difference (*p* > 0.05).

Of the isoprene derivatives, limonene (average value: 1.5 µg L^−1^) and citronellol (average value: 6.6 µg L^−1^) were present in the wine headspaces. Regarding the terpenoids, α-terpineol was detected in the grape samples, but was absent in the respective wines. Several studies are present in the literature [44,45,46] focused on terpenoid conversions, which require the presence of various microorganisms. The absence of α-terpineol in wine could therefore be linked to a biotransformation of this terpenoid during the wine making process.

The summary data for the VOCs revealed great variability among the wine samples. This variability may be explained by the different agronomic techniques employed and the diverse physical-chemical characteristics of the vineyards involved (Table 1). The wines richest in VOCS were NS4, followed by SS1 and NS2. We can observe that these grapes all belong to the same clone, CFC 13, and to the same rootstock. Again, the grapes of these three wine samples had a low yield and an important percentage of sand in the soil with respect to the silt and clay content. Our results agree with the observations reported by Sabon et al. [47] on Grenache wines obtained from different terroirs in the Rhone valley: primarily, that an increase in the concentration of volatile compounds correlates with early maturation, which occurs in the warmest soils. In fact, in our experiment, the SS1 site is certainly the most southerly and warmest, while the NS4 and NS2 sites are the closest to the sea and characterized by sandy soils, and thus characterized by warm soils.

### 3.4. Sensory Analysis 

**Figure 1:**
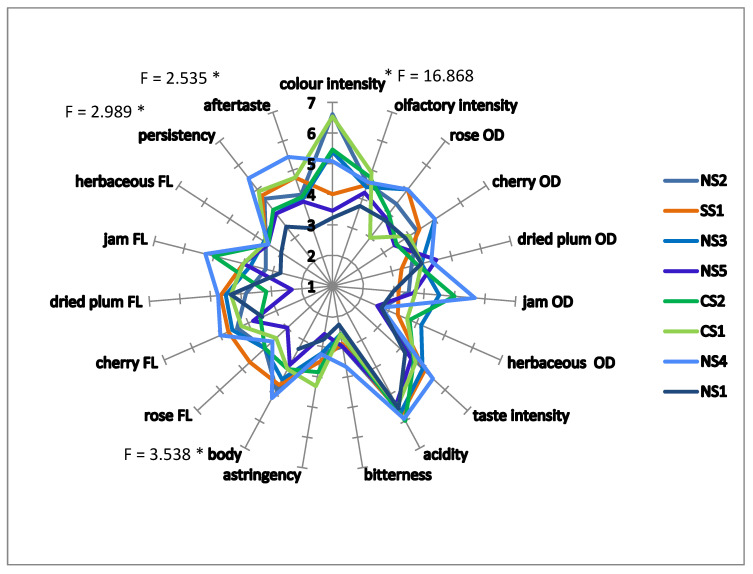
Graphical representation of the sensory profile of Cannonau wine samples. Values indicate the means for each attribute. The F values represent significant differences between the samples in relation to the individual attributes marked with the asterisk * (*p* < 0.05). NS1-5 = north Sardinian wines; CS1-2 = central Sardinian wines; SS1 = south Sardinian wines. OD represents olfactory attributes, while sensory attributes followed by FL (flavor) represent flavor attributes.

Data obtained from one-way ANOVA showed significant differences (*p* < 0.05) among the eight wine samples for the following attributes: color intensity, body, aftertaste, and persistency (Figure 1). More specifically, wine NS1 was rated to have the lowest color intensity, a scarce body, to be less persistent, and to have a low aftertaste. In relation to agronomic characteristics, this vineyard has the highest yield per vine with respect to all the other samples. This wine was followed by NS3 and NS5. Both wines received a low rating by the panel in relation to persistence and aftertaste. The NS4 sample reached the highest rating in relation to body, persistence, and aftertaste. It is important to underline the fact that this wine, as reported above, was characterized by the highest concentration of VOCs compounds. The CS1 and CS2 wines are characterized by a good color intensity, which was more intense, but not significantly more than the remaining samples. They also showed the highest astringency with respect to the other samples, although, once again, the difference was not statistically significant. The SS1 sample was the least herbaceous and was characterized by a good scent of rose.

**Figure 2:**
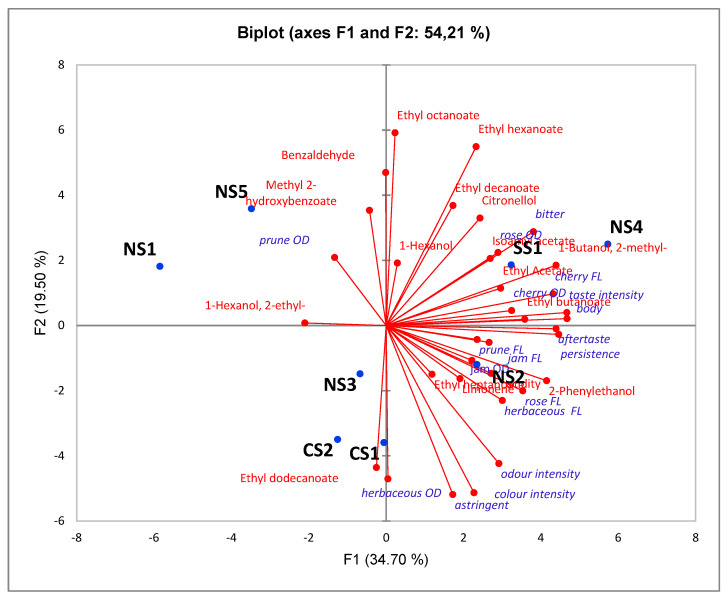
Principal component analysis (PCA) biplot for volatile organic compounds (VOCs) detected by GC-MS and sensory attributes. NS1-5 wines = north Sardinia; CS1-2 wines = central Sardinia; SS1 wine = south Sardinia. Sensory attributes followed by OD represent olfactory attributes, while sensory attributes followed by FL represent flavor attributes.

The quantitative data relating to wine VOCs and sensory data were subjected to principal component analysis in order to extract the most relevant information [32]. As reported in Figure 2, the PC1 explained 34.7% of the total variance, while PC2 explained 19.5%. In the bi-plot, three groups were evident. The first group was represented by samples NS1 and NS5, the second by CS1, CS2, and NS3, and the third group included SS1, NS2, and NS4 samples. The latter group was characterized by the highest VOC content, as reported above, and corresponded with the highest sensory attribute ratings. This group has in common the type of clone, CFC13, and the same rootstock, 1103P. With regard to the second group, formed by CS1, CS2 and NS3 samples, it can be observed that these wines derive from local selections. The first group, composed by NS1 and NS5, is characterized by wines obtained from different clones and different rootstocks, although they are situated in the same quarter of the biplot (Figure 2). Regarding the influence of the choice of rootstocks on the aroma compounds content, the literature is still scarce. However, similar to our results, differences induced by different rootstocks on aroma compounds were observed in potted Sauvignon Blanc and Cabernet-Sauvignon grapevines [48] and in wines obtained from cv. Albarin negro grown in the Asturias [49]. In our case, differences in the VOC content could be better explained by the type of clone. In fact, clones that confer less vigor to the grapevine, such as the CFC13, could promote, directly or indirectly, the synthesis of secondary metabolites.

In the bi-plot, the samples NS4, SS1, and NS2 were correlated to the medium-chain fatty acid esters: ethyl-butanoate, -hexanoate, -octanoate, and -decanoate, meaning that these wines contained high concentrations of these aromas in comparison with the other samples. This could have increased the fruity and floral notes perceived by the panel, confirming our findings reported above in relation to the VOC content for these wines.

The wines NS1 and NS5, in comparison with the other samples, were characterized by hints of prune, which seems to be correlated with the presence of benzaldehyde, methyl 2-hydroxybenzoate, and 2-ethyl-1-hexanol (equivalent sensory terms: bitter almond, sweet, and mild floral, respectively).

The second group contained samples characterized by the attributes herbaceous, astringent, and a high color intensity. We can also observe that of the esters, only the ethyl dodecanoate, attributing tropical fruit notes, characterized this group.

By performing the Pearson’s correlation test (*p* < 0.05) to assess for correlations between the sensory attributes and the volatile compounds, we revealed a high correlation (r = 0.753) between benzaldehyde and prune odor attributes, and between the content of 2-phenylethanol and odor intensity (r = 0.734). It is interesting to emphasize the role of 2-methyl-1-butanol in the sensory perception of wine in the mouth, as it showed high coefficient correlations with taste intensity, bitterness, body, cherry flavor, persistence, and aftertaste (with r ranging from 0.73 to 0.86). This molecule is known to be a compound that increases wine complexity [50]. A similar result was also observed for 2-phenylethanol, which increased taste intensity, astringency, cherry flavor, persistence, and aftertaste (with r ranging from 0.71 to 0.89).

Finally, PLSR analysis showed, for this set of wines, according to the loading weights and the regression coefficients, that 2-methyl-1-butanol and 2-phenylethanol can be considered the best predictors of wine sensory characteristics like taste intensity, bitterness, cherry flavor, body, persistence and aftertaste. The positive correlation of 2-phenylethanol and 3-methyl-1-butanol with the aromatic intensity of red wines and ripe fruit descriptors was also observed in other papers [51].

## 4. Conclusions

In conclusion, the results obtained showed a great variability among the Cannonau grape samples collected in different vineyards of Sardinia but the general VOC fingerprint of Cannonau grape skin and juice was very similar for all the samples studied. With reference to the wines, a predominance of yeast-derived compounds in the headspace was observed. The main volatile compounds found in the headspace were two isomers of methyl butanol (2-methyl-1-butanol and 3-methyl-1-butanol) and 2-phenylethanol, that showed an interesting relationship with the sensory attributes. It is important to observe how the standardization of the enological practices permitted us to explore this relationship in more detail. Nevertheless, to identify the molecules mainly linked to the wine sensory descriptors, further studies will be needed due to the complexity of the analysis of aromatic profiles from the analytical standpoint and to the substantial interactions among the aroma molecules.

## Figures and Tables

**Table 1:** Agronomic and physical-chemical characteristics of grapes and vineyards in the different locations

	Parameter	Unit	VINEYARDS							
			North					Centre		South
			NS1	NS2	NS3	NS4	NS5	CS1	CS2	SS1
**agronomic characteristics**	Altitude	m	40	120	40	50	260	220	220	40
	Yield	tons	152	76	65	91	119	98	106	109
	Yield/vine	kg	4.47	2.28	1.86	2.46	3.15	2.11	2.36	2.08
	Pruning		Spur cordon	Cane	Spur cordon	Cane	Cane	Cane	Spur cordon	Spur cordon
	Rootstock		1103 P	1103 P	Rupestris du lot	1103 P	140 R	1103 P	1103 P	1103 P
	Clone/biotype		Cfc 13	Cfc 13	Local selection	Cfc 13	Capvs 1	Local selection	Local selection	Cfc 13
	Irrigation		yes	yes	no	yes	yes	no	no	yes
**physico-chemical characteristics**	Sand	%	40.5	89.3	52	60.3	78.1	55	61	53.5
	Silt	%	23.9	2.2	35.5	18.5	5.1	20	18	7.5
	Clay	%	35.6	8.5	12.5	21.2	16.8	25	21	39
	pH	(H_2_O)	7.3	7.7	7.2	8.4	8.7	6.9	6.8	8.1
	Organic carbon	g/kg	4.9	2.8	0.73	2.3	2.7	0.96	1.26	2.5
	Organic matter	g/kg	14	7	1.26	5	16	0.8	0.7	4.4
	Active limestone	g/kg	8	6	9	22	53	2	1.2	42
	Total limestone	g/kg	26	14	23	95	166	11	9	122
	Total nitrogen	g/kg	0.7	0.6	0.10	0.7	0.51	0.6	0.9	0.56
	Base exchange	meq/100 g	16	10.4	18.1	8.6	30.2	6.70	5.2	15.4

NS, CS and SS mean north Sardinia area (5 samples), central Sardinia area (2 samples) and south Sardinia area (1 sample), respectively.

**Table 2:** Volatile compounds detected in the skins and juices of Cannonau grape samples from Sardinia and calibration equations used for the quantification of the volatile compounds.

Compound	Skin F	Skin B	Juice F	Juice B	Calibration Equation	R^2^	Linearity Range (µg/mL)	RI
2-Methyl-2-butanol *	x	x	x	x	2-methyl-1-butanol			1022
5-Isopropyl-3,3-dimethyl-2-methylene-2,3-dihydrofuran	x	x	x	x	nq			1026
Butyl acetate	x	x	x	x	y = 0.938x − 0.0037	0.9991	0.002–2.38	1089
Hexanal	x		x		y = 0.755x − 0.0064	0.9995	0.002–2.4	1103
1-Methoxy-2-propanol			x		nq			1143
Butanol	x	x	x	x	y = 0.1246x + 0.0174	0.9969	0.008–0.84	1152
Pent-3-en-2-ol *	x	x	x	x	(Z)-3-Hexen-1-ol,			1178
2-Methyl-1-butanol					y = 1.4888x + 0.0053	0.9995	0.0002–0.238	1213
n-Pentanol *	x				Hexanol			1215
Limonene *	x	x	x	x	α-Terpineol			1217
1-Methoxypropan-2-yl acetate	x	x	x		nq			1233
(*E*)-Hex-2-enal	x		x		y = 1.391x + 0.0047	0.9986	0.061–61.46	1241
3-Octanone	x	x	x	x	nq			1269
2-Methyloctan-2-ol	x	x	x	x	nq			1318
Cyclohexanone	x	x	x	x	nq			1320
1-Butoxypropan-2-ol	x	x	x	x	nq			1346
Hexanol	x		x		y = 2.092x − 0.0094	0.9987	0.001–0.986	1352
(*Z*)-Hex-3-en-1-ol	x		x		y = 1.816x − 0.0169	0.9986	0.026–26.2	1372
(*E*)-Hex-3-en-1-ol *	x		x		(*Z*)-hex-3-en-1-ol			1385
Nonanal	x	x		x	y = 1.256x − 0.0046	0.9993	0.009–9.48	1403
(*E*)-Hex-2-en-1-ol *	x		x		(*Z*)-hex-3-en-1-ol			1403
2-Butoxyethan-1-ol	x	x	x	x	nq			1407
Terpenoid *	x	x	x	x	α-Terpineol			1424
2-Ethyl-1-hexanol	x	x	x	x	y = 2.114x + 0.002	0.999	0.013–12.56	1479
Benzaldehyde	x	x	x	x	y = 1.86x − 0.0122	0.9985	0.003–2.52	1547
Isophorone	x	x	x	x	nq			1622
Acetophenone	x	x	x	x	y = 1.734x − 0.0012	0.9968	0.0009–0.94	1672
α-Terpineol	x	x	x	x	y = 2.187x + 0.00009	0.9986	0.001–1.42	1695
Naphthalene	x	x	x	x	Nq			1764
Hex-2-enoic acid	x	x	x	x	nq			1818
2-Ethyl-3-hydroxyhexyl isobutyrate	x	x	x	x	nq			1848
Benzyl alcohol	x	x	x	x	y = 1.93x + 0.0267	0.9987	0.003–0.659	1862
Methylnaphthalene (not identified isomer)	x	x	x	x	nq			1868
2-Phenylethanol	x	x	x		y = 2.02x + 0.0002	0.9986	0.003–2.86	1894
Methylnaphthalene (not identified isomer)	x	x	x	x	nq			1901
Benzo[*d*]thiazole	x	x	x	x	nq			1962
Dimethylnapthalene (not identified isomer)	x	x	x	x	nq			1969
Dimethylnapthalene (not identified isomer)	x	x	x	x	nq			2004

Skin F and Juice F, free compounds from skin and juice, respectively; Skin B and Juice B, glycosylated compounds from skin and juice, respectively. RI, experimental linear retention indexes calculated on a WF-Wax column; nq, not quantified; * quantified with a calibration curve of a compound of the same chemical class as reported in the “calibration equation” column.

**Table 3:** Range and average levels of volatile-free aroma compounds in the skin and pulp of Cannonau grapes collected in Sardinia.

	SKIN FREE (µg kg^−1^)							JUICE FREE (µg kg^−1^)						
	NS			CS			SS	NS			CS			SS
	Max	Min	Mean	Max	Min	Mean	Mean	Max	Min	Mean	Max	Min	Mean	Mean
2-Methyl-2-butanol *	20.7	14.6	17.5 ^ns^	24.0	12.8	19.0 ^ns^	16.9	21.9	14.2	18.4 ^ns^	19.7	13.4	16.0 ^ns^	15.8
Butyl acetate	40.8	19.9	33.0 ^ns^	51.2	17.0	32.9 ^ns^	38.2	45.5	4.7	26.6 ^ns^	42.8	19.9	29.3 ^ns^	41.4
Hexanal	643.5	238.7	388.0 ^ns^	467.3	131.0	303.5 ^ns^	408.7	813.9	300.7	536.9 ^ns^	658.1	101.5	344.5 ^ns^	636.8
Pent-3-en-2-ol *	79.7	57.0	67.5 ^ns^	89.1	50.1	73.1 ^ns^	66.1	61.4	21.0	42.6 ^ns^	49.4	36.5	42.3 ^ns^	36.8
n-Pentanol *	17.0	8.9	11.3 ^ns^	13.2	6.0	10.4 ^ns^	11.9	nd	nd	nd	nd	nd	nd	nd
Limonene *	4.9	0.0	1.3 ^ns^	4.2	0.04	1.5 ^ns^	1.5	5.7	0.04	1.9 ^ns^	13.5	0.04	3.8 ^ns^	16.0
(*E*)-Hex-2-enal	875.2	377.8	518.9 ^ns^	808.8	230.5	575.8 ^ns^	544.8	571.6	311.0	424.8 ^ns^	543.4	156.6	401.8 ^ns^	855.6
Hexanol	41.1	13.3	32.9 ^ns^	39.4	14.8	29.8 ^ns^	12.7	113.7	82.9	92.8 ^ns^	175.5	11.2	87.4 ^ns^	106.8
(*Z*)-Hex-3-en-1-ol	16.4	8.8	12.6 ^ns^	27.5	12.3	19.9 ^ns^	nd	18.0	9.3	11.7 ^ns^	105.2	9.3	31.8 ^ns^	9.3
Nonanal	158.2	70.7	116.6 ^ns^	179.0	58.1	103.8 ^ns^	133.6	nd	nd	nd	nd	nd	nd	nd
(*E*)-Hex-2-en-1-ol *	nd	nd	nd	nd	nd	nd	nd	149.4	63.2	109.4 ^ns^	186.1	75.2	126.6 ^ns^	145.5
Terpenoid *	4.8	0.0	1.7 ^ns^	1.9	0.01	0.7 ^ns^	0.01	2.3	0.04	0.77 ^b^	14.3	0.04	6.1 ^a^	0.04
2-Ethyl-1-hexanol	90.6	36.5	74.0 ^ns^	107.4	35.7	63.7 ^ns^	82.8	102.3	5.4	60.0 ^ns^	111.3	34.8	67.3 ^ns^	83.2
Benzaldehyde	147.2	18.6	111.2 ^ns^	174.8	16.5	78.3 ^ns^	134.6	182.4	12.7	103.6 ^ns^	158.7	14.1	89.7 ^ns^	159.4
Acetophenone	8.2	0.7	4.0 ^ns^	10.9	0.7	6.0 ^ns^	7.4	12.5	0.7	7.2 ^ns^	12.6	7.2	9.2 ^ns^	7.4
α-Terpineol	15.7	3.9	9.0 ^ns^	15.6	2.6	7.0 ^ns^	9.2	14.6	0.04	8.3 ^ns^	13.2	0.04	5.4 ^ns^	10.8
Benzyl alcohol	238.1	58.1	112.5 ^a^	88.7	25.0	54.8 ^b^	68.9	106.0	44.4	67.8 ^a^	72.6	7.9	35.9 ^b^	81.8
2-Phenylethanol	34.3	26.8	31.5 ^a^	32.4	12.5	24.9 ^b^	22.7	19.8	7.7	13.4 ^ns^	23.5	4.6	15.0 ^ns^	13.6
Total VOCs			1543.5			1405.1	1560.0			1526.2			1312.1	2220.2

NS: samples collected in north Sardinia area (*n* = 6); CS: samples collected in central Sardinia area (*n* = 7); SS: sample collected in south Sardinia area (*n* = 1). nd: not detected; tr: trace; ^ns^: not significant. Different letters in the row mean significant differences among the areas (*p* < 0.05). Data are the mean of three replicates. *: quantified with a calibration curve of a compound of the same chemical class as reported in Table 2.

**Table 4:** Range and average levels of volatile glyco-conjugated aroma compounds in the skin and pulp of Cannonau grapes collected in Sardinia.

	SKIN BND (µg kg^−1^)							JUICE BND(µg kg^−1^)						
	NS			CS			SS	NS			CS			SS
	max	min	mean	max	min	mean	mean	max	min	mean	max	min	mean	mean
2-Methyl-2-butanol *	47.0	17.9	26.5 ^ns^	49.0	15.3	30.0 ^ns^	22.4	30.6	11.0	21.3 ^ns^	42.1	13.8	25.2 ^ns^	17.3
Butyl acetate	36.8	0.00	20.9 ^ns^	52.4	4.3	30.8 ^ns^	26.7	50.0	4.7	23.3 ^ns^	46.2	7.5	26.3 ^ns^	43.4
Butanol	19.0	0.00	13.3 ^ns^	28.0	6.1	16.8 ^ns^	15.2	18.9	6.8	14.1 ^ns^	31.8	11.0	17.5 ^ns^	14.1
Pent-3-en-2-ol *	93.7	52.1	79.6 ^ns^	124.2	15.0	79.9 ^ns^	84.4	57.7	10.1	44.7 ^ns^	65.5	27.9	48.2 ^ns^	38.3
Limonene *	4.8	0.00	2.1 ^ns^	5.5	0.3	3.4 ^ns^	0.5	7.2	0.2	2.8 ^ns^	6.3	0.9	3.4 ^ns^	3.4
Nonanal	162.1	42.0	105.1 ^ns^	166.6	8.3	98.9 ^ns^	39.9	150.7	10.8	71.9 ^ns^	149.8	22.3	90.6 ^ns^	127.6
Terpenoid *	12.0	0.00	5.7 ^ns^	16.3	0.9	7.7 ^ns^	0.00	9.5	0.4	4.7 ^ns^	15.4	2.4	8.1 ^ns^	tr
2-Ethyl-1-hexanol	147.7	37.2	77.0 ^ns^	140.3	3.6	84.0 ^ns^	38.4	111.5	4.5	57.2 ^ns^	129.0	22.2	78.6 ^ns^	86.3
Benzaldehyde	246.2	19.2	106.2 ^ns^	305.5	11.8	178.3 ^ns^	17.4	242.8	12.7	105.2 ^ns^	261.8	17.5	137.5 ^ns^	196.2
Acetophenone	13.3	4.6	7.9 ^ns^	21.1	1.3	12.5 ^ns^	6.4	11.3	1.8	6.7 ^ns^	14.5	4.5	9.9 ^ns^	9.1
α-Terpineol	17.1	3.0	8.8 ^ns^	20.0	0.00	10.8 ^ns^	3.6	13.2	0.9	6.6 ^ns^	14.6	1.9	8.8 ^ns^	13.4
Benzyl alcohol	216.9	9.4	80.9 ^ns^	282.3	5.3	166.7 ^ns^	10.2	282.3	3.9	121.6 ^ns^	272.7	7.6	126.0 ^ns^	190.2
2-Phenylethanol	6.9	0.00	3.5 ^ns^	10.3	0.2	4.8 ^ns^	nd	nd	nd	nd	nd	nd	nd	nd
Total VOCs			537.5			724.6	265.1			480.1			580.1	739.3

NS: samples collected in north Sardinia area (*n* = 6); CS: samples collected in central Sardinia area (*n* = 7); SS: sample collected in south Sardinia area (*n* = 1). nd: not detected; tr: trace; ^ns^: not significant. Data are the mean of three replicates. *: quantified with a calibration curve of a compound of the same chemical class as reported in Table 2.

**Table 5:** Concentration of the main volatile organic compounds in Cannonau wines from the different areas.

Compound	NS1		NS2		NS3		NS4		NS5		CS1		CS2		SS1	
	µg L^−1^	SD	µg L^−1^	SD	µg L^−1^	SD	µg L^−1^	SD	µg L^−1^	SD	µg L^−1^	SD	µg L^−1^	SD	µg L^−1^	SD
1-Ethoxy-1-methoxyethane *																
Ethyl Acetate	24,255c	36	33,049a	2944	18,088d	124	28,469b	1223	20,444d	1716	21,127d	774	25,295c	385	33,049a	121
Ethyl butyrate	110.5e	5.1	481.9a	45.8	163.5de	8.7	328.6b	19.3	234.8cd	48.2	294.6bc	44.6	160.2de	17.2	345.2b	35.1
2-Methyl-1-propanol *																
Isoamyl acetate	158.4ab	30.4	118.7bc	7.2	63.5	20.4	221.3a	7.7	38.9c	7.2	108.3bc	83.4	82.4bc	70.2	161.4ab	6.3
Limonene	1.48ab	0.03	1.64a	0.12	1.51ab	0.14	1.49ab	0.01	1.44b	0.03	1.47b	0.03	1.48ab	0.01	1.50ab	0.02
2-Methyl-1-butanol **	92,310e	4405	159,247b	7786	98,603de	6420	181,830a	6218	122,528c	17,625	124,113c	11,918	115,444cd	2971	163,498ab	1960
Ethyl hexanoate	704.7cd	18.4	724.9bc	1.4	664.6	21.7	779.4ab	4.9	743.4abc	52.5	655.0cd	15.2	625.8d	0.6	784.1a	29.2
3-Octanone *																
Styrene *																
Ethyl heptanoate	tr		0.47a	0.38	0.13b	0.07	tr		tr		tr		0.09b	0.09	tr	
Hexanol	897bc	41	1536a	31	734c	41	1041b	48	1581a	302	1089b	20	936bc	2	1041b	99
Ethyl octanoate	154.9a	1.3	111.9b	2.4	124.9b	12.1	154.0a	7.0	152.2a	16.3	110.5b	7.3	107.9b	2.5	168.8a	20.1
Acetic acid *																
2-Ethyl-1-hexanol	1.8	0.4	tr		8.8	0.7	tr		4.7	0.5	tr		1.8	0.2	tr	
Ethyl nonanoate *																
Benzaldehyde	39.5c	10.0	31.1cd	11.3	12.4d	11.4	145.6b	4.0	208.0a	9.3	13.4d	9.3	31.4cd	8.9	28.2cd	15.3
Methyl decanoate *																
Ethyl decanoate	41.8ns	4.6	41.5	3.6	40.8	4.6	67.3	0.8	46.0	7.4	30.4	4.6	33.7	1.7	31.5	58.2
Nonanol *																
Isoamyl octanoate *																
Diethyl succinate *																
Ethyl dec-9-enoate *																
3-(Methylthio)-1-propanol *																
Ethyl undecanoate *																
Citronellol	4.2b	0.1	2.6b	1.1	3.0b	1.6	11.3ab	5.0	5.0b	0.6	2.1b	0.2	0.7b	0.4	27.2a	23.9
Methyl dodecanoate *																
Methyl 2-hydroxybenzoate	0.8bc	0.7	0.1c	0.1	3.3b	1.6	tr		13.0a	1.8	2.1bc	1.0	tr		11.9a	1.8
Phenethyl acetate *																
Ethyl dodecanoate	0.35abc	0.06	0.51ab	0.42	0.16bc	0.11	0.26abc	0.12	0.09bc	0.08	0.51ab	0.10	0.54a	0.03	tr	
*β*-Damascenone *																
2-Phenylethanol	45,352c	2942	55,954b	3236	52,787b	2360	66,077a	5436	44,126c	943	64,783a	987	56,035b	1196	66,107a	3311
Ethyl tetradecanoate	tr		tr		tr		tr		tr		tr		tr		tr	
Ethyl pentadecanoate *																
Methyl hexadecanoate *																
Ethyl hexadecanoate *																
Total VOCs	164,032		251,301		171,299		279,126		190,126		212,330		198,756		265,255	

NS1–5 wines obtained by grapes from northern Sardinia. CS1–2 wines obtained by grapes from central Sardinia. SS1 wines obtained by grapes form southern Sardinia. Results are expressed as µg L^−1^ ± Standard Deviation (*n* = 2). Different letters for each row mean significant differences among the samples (*p* < 0.05); * Detected but not quantified; ** overlapped with 3-methyl-1-buthanol and quantified as 2-methyl-1-butanol equivalent; ns: not significant; tr: trace.

## Data Availability

Not applicable.

## Supplementary Materials

The following are available online at https://www.mdpi.com/2304-8158/10/1/101/s1, Figure S1: Response surface plots of the predicted sum of analytes:internal standard area ratios for: (A) Alcohols; (B) Esters; (C) Acids and (D) Terpens. Table S1: Main compounds detected in the headspace of Cannonau wines from Sardinia and calibration equations used for quantification of compounds. RI, experimental linear retention indexes calculated on an WF-Wax column. nq, not quantified., Table S2: Central Composition Design Matrix of variables optimized for the Solid Phase MicroExtraction.
