# Supplementary material for: Grape and Wine Composition in Vitis vinifera L. cv. Cannonau Explored by GC-MS and Sensory Analysis
Source: Foods. 2021 Jan 6;10(1):101. doi: 10.3390/foods10010101 (PMC7825112; doi:10.3390/foods10010101)
Supplement: Supplementary file 1 [file foods-10-00101-s001.pdf]

Table S1. Main compounds detected in the headspace of Cannonau wines from Sardinia and calibration equations used for quantification of compounds. RI, experimental linear retention indexes calculated on an WF-Wax column. nq, not quantified.

Compound	RI	Calibration equation	R^2	Linearity range (g mL^{-1})
1-Ethoxy-1-methoxy-ethane	835	nq		
Ethyl Acetate	882	y=0.0321 − 0.0027	0.9993	1.8–37
Ethyl butanoate	1076	y=0.2721 − 0.0149	0.9973	0.014–2.11
2-Methyl-1-propanol	1095	nq		
Isoamyl acetate	1105	y=0.4346x − 0.0388	0.9562	0.009–1.305
Limonene	1182	y=15.918x − 0.0157	0.9792	0.001–0.07
2-Methyl-1-butanol ovlp 3-Methyl-	1200	y=0.0221x + 0.0561	0.996	0.4–180
Ethyl hexanoate	1215	y=0.09258x + 0.281	0.996	0.052–7.83
3-Octanone	1245	nq		
Styrene	1256	nq		
Ethyl heptanoate	1315	y=7.959x + 0.0129	0.9978	9E-04–0.045
1-Hexanol	1335	y=0.088 + 0.0443	0.9954	0.018–2.715
Ethyl octanoate	1419	y=9.0921x + 0.1693	0.9999	0.002–2.955
Acetic acid	1471	nq		
2-Ethyl-1-hexanol	1522	y=71.1898x + 0.009	0.9941	0.001–0.055
Ethyl nonanoate	1519	nq		
Benzaldehyde	1547	y=1.297x + 0.0185	0.9982	0.008–0.395
Methyl decanoate		nq		
Ethyl decanoate	1628	y=22.818x + 0.2955	0.9995	0.002–3.24
Nonanol	1643	nq		
Isoamyl octanoate	1648	nq		
Diethyl succinate	1735	nq		
Ethyl 9-decenoate	1743	nq		
3-(Methylthio)-1-propanol	1768	nq		
Ethyl undecanoate	1776	nq		
Citronellol	1795	y=0.7479x + 0.00091	0.9948	0.001–0.23
Methyl dodecanoate	1803	nq		
Methyl salicylate	1810	y=2.7173x + 0.0099	0.9995	0.0001–0.08
Phenethyl acetate	1812	nq		
Ethyl dodecanoate	1814	y=80.923x + 0.123	0.9958	2E-05–0.022
-damascenone	1820	nq		
2-Phenylethanol	1846	y=0.0257x + 0.0239	0.9995	120.4–10.04
Ethyl tetradecanoate	1890	y=51.212x + 0.1488	0.9997	2E-04–2.385
Ethyl pentadecanoate	1932	nq		
Methyl hexadecanoate	1962	nq		
Ethyl hexadecanoate	1979	nq		

Table S2. Central Composition Design Matrix of variables optimized for the Solid Phase MicroExtraction.

**Run**	**Extraction time**		**Extraction temperature**	
	Coded level	(min)	Coded level	(°C)
	0			
1	0	35	0	50
2	0	40	1	60
3	0	35	1.41	64
4	−1.41	28	0	50
5	+1.41	42	0	50
6	0	35	0	50
7	0	35	0	50
8	−1	30	−1	40
9	1	40	−1	40
10	−1	30	1	60
11	0	35	0	50
12	0	35	−1.41	36
13	0	35	0	50

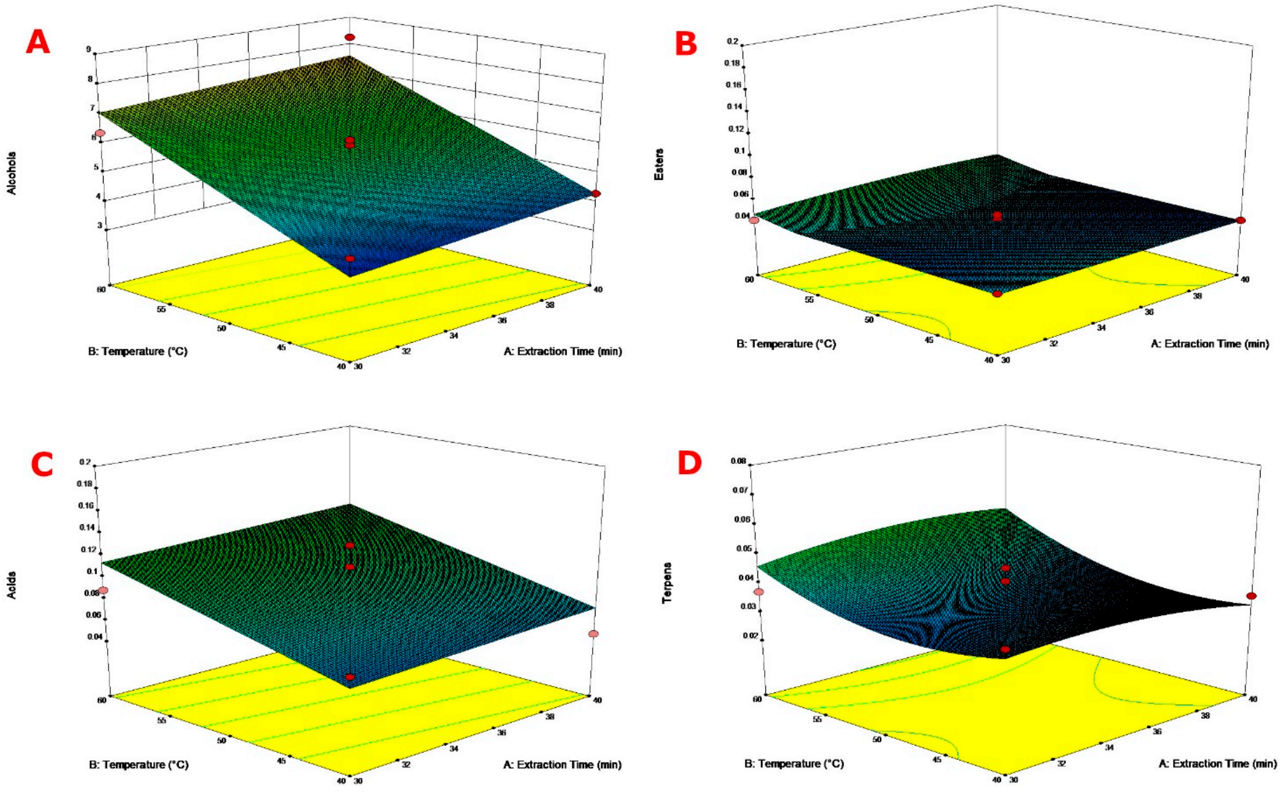

Figure S1. Response surface plots of the predicted sum of analytes:internal standard area ratios for: A) Alcohols; B) Esters; C) Acids and D) Terpens.
